# Interfacial Phenomena at the Interface in the System «Carbon Primary Materials-Water Solutions of Surfactants» for Cement Materials

**DOI:** 10.3390/ma15020556

**Published:** 2022-01-12

**Authors:** Svetlana Shekhovtsova, Evgenii Korolev

**Affiliations:** 1 Department of Building Materials Science, Moscow State University of Civil Engineering, RU 129337 Moscow, Russia; 2 Scientific and Educational Center “Nanomaterials and Nanotechnologies”, Institute of Construction and Architecture, Moscow State University of Civil Engineering, RU 129337 Moscow, Russia; korolev@nocnt.ru

**Keywords:** concrete, carbon nanotubes, surfactants, adsorption, surface activity, diffuse layer, two-dimensional pressure, peptization, stabilization, strength

## Abstract

The formation of sustainable concrete is directly relaed to the intensity of the processes occurring at the interface of phases. The study of the surface properties of CNPLUS carbon nanotubes in solutions of various plasticizers was carried out by measuring and calculating adsorption. The applicability of the adsorption value is for forecasting both the efficiency of dispersion and aggregative and sedimentative stability of the obtained dispersion systems. It was stated that two-dimensional pressure arising at the interface of adsorption layers in the dispersive medium with the surfactant Tensafor 2553.2 J/m^2^ is sufficient to overcome adhesive strength on a small area of the localized contact of carbon nanoparticles CNPLUS, which explains the peptization and stabilization of the particles’ surface. It was established that full stabilization of nanoparticles in the aqueous dispersive medium could be ensured only by means of soap-like surfactants, with the compound potassium naphthalene sulfonate (Tensafor). It ensures formation of the micelle-like structure in coagulation layers that forms a structural and mechanical barrier with the external hydrophilic surface. This leads to the increase in the ultimate tensile strength of the concrete grout specimens by 38%.

## 1. Introduction

At present, materials science increasingly frequently considers the main properties of substances, phenomena and processes partaking in the formation of a construction composite at the nanoscale. Due to their unique mechanical, thermal and optical properties, carbon nanomaterials are of great interest not only as a scientific research object but also as an object of technology providing control over structure formation of a composition, creating new properties and the quality level [1,2,3]. By introducing small concentrations of carbon materials into the cement matrix or their synthesis in the process of composite manufacturing, it becomes possible to exercise the directed control over formation of products’ performance properties [4,5,6,7,8,9]. In the field of civil engineering, many studies have been conducted on the use of CNTs for modified concrete and cement structures. Stynoski et al. [10] reported an increase of 11% in maximum stress and an increase of 11.4% in Young’s modulus of elasticity with the addition of 0.125 wt% CNTs. Xu et al. [11] recorded an increase of about 22% in the compressive strength of cement with the addition of 0.1 wt% MWCNT, and Bharj et al. [12] recorded similar increases in cement compressive strength with the addition of 0.1 wt% CNTs. Other studies have reported a dramatic improvement of mechanical and flexural properties of CNT-reinforced cement [12,13,14]. Among these reports, an appreciable increase in tensile strength of up to 260% was observed in a concrete structure with 0.2 wt% MWCNT as additives [12]. Musso et al. [15] compared three different modified 0.5 wt% MWCNT in concrete as pristine, annealed and carboxyl-group functionalized MWCNTs. The functionalized MWCNTs showed dramatic decreases of compressive strength (from 103 to 18 KN) and rupture modulus (from 7.5 to 3 MPa), while other two CNT types showed partial increases in compressive strength (from 103 to 118 KN) and rupture modulus (from 7.5 to 8 MPa). Impermanent dispersion and particle flocculation has been a fundamental problem reported by several researchers regarding CNT or carbon-nano-fiber (CNF) reinforced concrete structures [16,17].

It is important to note that in both the development of new modifying materials on the base of primary carbon materials and the application of existing additives, their comprehensive study is a must [1,2,3]. However, regardless a dispersive system in consideration, the researchers come across the common issue related to ensuring effective dispersion and even volumetric distribution of primary carbon materials in both the carrier and composite matrix due to spontaneous aggregation specific to carbon nanomaterials [3]. There is a way of incorporating nanomaterials into a cementitious composite in which the nanomaterials such as carbon nanofiber (CNF) and graphene oxide are coated on the fiber instead of direct addition into the dry mixture [18,19]. He et al. managed to attach the CNF on the PE fiber to strengthen the interfacial transition zone (ITZ) and enhance the fiber/matrix frictional bond of the strain hardening ultra-high-performance concrete (SHUHPC). The SHUHPC with CNF coated PE fiber achieved 15% and 20% increase in tensile strength and strain capacity, respectively. Research has been undertaken previously on Portland cement–CNT paste and mortar nanocomposites, showing significant enhancements to the mechanical and durability performance of the material, although the degree of enhancement varies between studies due to CNT dispersion challenges. Extensive reviews on these paste and mortar composites can be found elsewhere [20,21,22,23]. However, while some studies have reported on the mechanical properties [24,25] and durability [26,27] of CNT-reinforced concrete composites (CNTRC), scaling up from pastes and mortars to feasible real-world practical, safe and economical utilization of CNTs in concrete construction applications remains challenging. There is an effective way that nanoscale modifiers are introduced into the composite matrix in the form of colloid dispersive system with a plasticizer. In present days, effective techniques for the dispersion of CNT-reinforced cement mixtures include the use of surfactants and ultrasonic energy [28,29,30]. Zou et al. [21] focused on the impact of ultrasonic energy on carbon nanotube dispersion, and the well dispersed impacts on the properties of CNT-modified cements [31]. For this purpose, apart from optimizing technological properties of a mortar or concrete mixture, it is required to select an appropriate plasticizer that enables to obtain good compatibility of a composite matrix and primary nanosized materials and achieve homogeneous modification of a composite matrix. The stability problems which may occur in the formation of cement systems are due to the occurring adsorption processes. However, these processes were investigated only in the “cement–surfactant” system [32,33,34,35,36,37,38]. In the present study, research of physical and chemical properties of dispersive systems with primary nanosized materials presents the well-known difficulties related to the appearance of so-called dimensional effects caused by the anomality of the particular particles’ characteristics as well as their auto-adhesion interaction [36,37,38]. The issues arising due to the intermediate position occupied by nanosized particles between molecules (atoms, ions) and macroscopic bodies in the building materials technology are solved by the methods applied in colloid chemistry [39].

The study of interfacial processes is reduced to the study of the nature of intermolecular interactions [40,41,42,43]. In turn, they determine the balanced behavior of pseudo-balanced systems, dynamic and static properties of liquid phases, the structure of amorphous compounds, etc. Adsorption is seen as one of the manifestations of intermolecular interactions. Dispersive systems with cluster nanomaterials are of special interest.

In the given dimensional scale, a substance possesses specific surface-excessive properties and, at the same time, the properties of a volumetric phase that enables to exercise control over structure formation of a material at a greater extent and enhance its performance characteristics significantly. From the perspectives of classical physics, it follows that due to particles size reduction, the increase in the specific surface area of a dispersive phase is observed which leads to the increase in the contribution of excessive surface energy into the total free energy of a system. One of the characteristics that allows evaluating the excess of surface energy is the adsorption value described by the Gibbs thermodynamic equation [44]:

(1) $$\Gamma =-\frac{\Delta \sigma }{\Delta C}\cdot \frac{C}{RT},$$

where Γ—adsorption, mol/m^2^; *C*—equilibrium concentration of the dissolved substance, mol/dm^3^; *R*—the universal gas constant (8314 J/mol·K); *T*—temperature, K; Δ*σ*—the change of superficial tension; Δ*C*—the change of concentration.

The present article is dedicated to the study of the surface properties of nano-dispersive particles—doped multi-walled carbon nanotubes CNPLUS in the solutions of various plasticizers applied for manufacturing structural construction composites.

The value of the plasticizer’s adsorption on primary carbon materials was taken as the base characteristic for their surface activity Γ*_f_*. Calculation of Gf was done on the base of experimental data on the change of superficial tension at the interface “colloid solution of a plasticizer with primary carbon material–air”.

In order to describe the dependency of superficial tension change of plasticizer’s aqueous solution on the concentration of primary carbon material, Shishkovsky’s empirical equation [45] was used, which is true for highly diluted solutions. In this case, the constant *A* is related to the value of maximum adsorption A = *RT*Γ_max_ and does not depend on the length of a hydro carbonic group of the surfactant’s molecule (provided the vertical orientation of diphilic molecules and their high density), whereas the constant *B* coincides with the value of surface activity from the Langmuir’s equation [46]. On the base of this, Shishkovky’s empirical equation takes the following form:

(2) $$\sigma =\sigma_{0}-B\ln\left(\frac{c}{A}+1\right),$$

where c—surfactant’s concentration; $\sigma_{0}$—the constant describing superficial tension of water; *B* and *A*—empirical constants.

Surface activity, i.e., the ability of a diluted substance to lower Gibbs’s surface energy on the given interface, is connected with the constants from Shishkovsky’s equation under the following dependency:

(3) $$-\frac{\Delta \sigma }{\Delta C}\cdot \frac{C}{\mathrm{RT}}=\frac{B}{\mathrm{RT}}\cdot \frac{\mathrm{AC}}{1+\frac{c}{A}},$$

having transformed which, we find the dependency describing the surfactant’s activity:

(4) $$\frac{\Delta \sigma }{\Delta C}=-\frac{B}{\left(1+\frac{c}{A}\right)}$$

when carbon nanosized materials are introduced into the surfactant’s solution, ultrasonic cavitation takes place, and the solid surface is capable to adsorb surfactant molecules, redistribution of their molecules in the system “surface-aqueous solution of a surfactant with carbon nanosized materials–air–solution–solid surface” will occur. This will lead to bringing down the concentration of the surfactant’s molecules on the solution surface and the formation of the adsorption layer on the particles of carbon nanosized materials. It is expected that during adsorption of surfactants on the solid surface, auto-adhesion strength would decrease and the dispersion intensity would increase. The effectiveness of dispersion is expected to increase in direct proportion to the growth of the surfactants’ adsorption value for carbon nanosized materials. It is obvious that lowering the concentration of surfactant’s molecules on the surface “aqueous solution–air” would lead to the increase in superficial tension according to the Gibbs equation. Superficial tension is a rather convenient property of solutions, which is accurately determined by experiments.

After redistribution (achievement of equilibrium), the surfactants’ concentration at the interface “aqueous solution of surfactants–air” can be calculated under the following formula:

(5) $$C_{x}=A\left(\exp\left(\frac{\sigma_{0}-\sigma_{x}}{B}\right)-1\right)$$

The value of adsorption activity of surfactants’ molecules at the interface “aqueous solution of surfactants–air” is equal to:

(6) Γ0=BA(1+C0A)·C0RT=BRT(1−(1exp(σ0−σ (C0)B)))

Having introduced carbon materials into the surfactant solution, the value of specific adsorption at the interface “aqueous solutions of surfactants–air” is determined under the formula:

(7) Γx=BA(1+CxA)·CxRT=BRT(1−(1exp(σ0−σx B)))

The value of surfactants’ adsorption (plasticizer’s molecules) will be equal to:

(8) $$\Gamma_{f}=\Gamma_{0}-\Gamma_{x}$$

it is expected that the higher the difference between $\Gamma_{0}$ and $\Gamma_{x}$, the more effective are the carbon nanosized materials, where the aggregate and sedimentative stability of the obtained dispersions would be ensured. This will ensure the formation of sustainable concrete.

## 2. Materials and Methods

### 2.1. Raw Materials and Characterization

In the article, plasticizers of anionic type on the base of polycarboxylic ethers ViscoCrete 2100, produced by Sika Canada Inc., being carboxylic polymers, were used as surfactants, as well as Tensafor NF Powder produced by Conquímica S.A. Colombian, being potassium naphthalene sulfonate. Table 1 summarizes the characteristics of the plasticizer as provided by the manufacturer.

**Figure 1:**
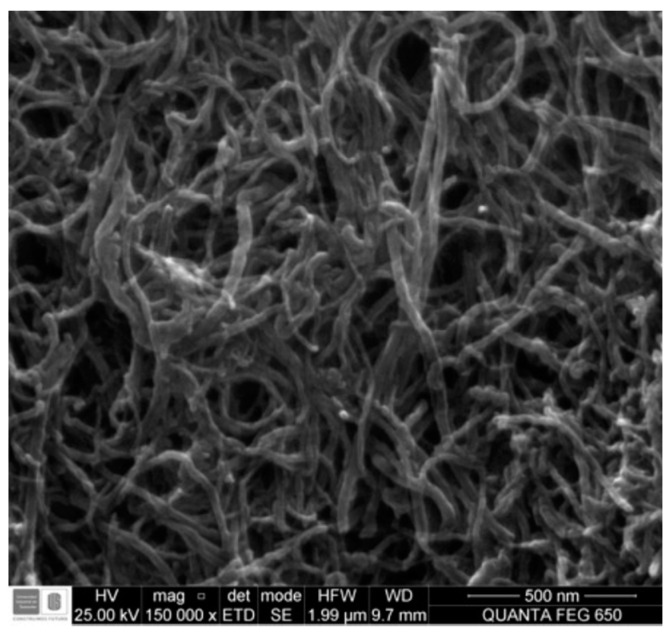
Morphology of the CNPLUS.

The carbon primary materials were functionalized multi-walled carbon nanotubes (MWNT), produced by Carbon Nanotubes (CNPLUS), USA. Table 2 and Figure 1 summarize the characteristics of the CNTs as provided by the manufacturer.

As a binder for concrete specimens, Ordinary Portland cement type I (CEM I 42.5 N) was used, produced by the “Eurocement Group”, bulk density (loose) 1020 kg/m^3^; as an aggregate—quartz fluvial sand (the name of the production site), with the bulk density 1.54 g/cm^3^, true specific gravity 2.65 g/cm^3^, and fineness modulus 2.43.

### 2.2. CNT Dispersion Methods

Surfactants were added to the water. After then, the suspension was mixed for 5 min and altered using a magnetic stirrer with a speed of 800 rpm/min. The carbon nanotubes were then added and mixed for some time using ultrasonic dip-type dispersion machine Vibra Cell, VCX750, emission power 700 Wt, with the amplitude of impact 75% and frequency 20 Hz. The percentage composition of a nano-modifier for these systems was taken in compliance with the requirements of a manufacturer and accounted for 0.05%.

### 2.3. Cementitious Composite Fabrication

Cementitious composites were prepared according to the EN 197-1:2011 and EN 196-1:1994 standards. Cementitious composites contained a dispersed aqueous solution with carbon nanotubes, ordinary Portland cement and quartz fluvial sand. The laboratory mixer was used for mixing. For samples cementitious composites with ViscoCrete 2100, a water-to-cement ratio of 0.5 was used. For samples cementitious composites with Tensafor NF powder, a water-to-cement ratio of 0.45 was used. To prepare the mortar mixture, cement (450 g) was placed in the mixer bowl. Then, different concentrations of carbon nanotubes were added to the cement, which were previously dispersed in 225 (202.5) mL of water. Afterwards, the required amount of quartz fluvial sand (1350 g) was added into the mixing bowl.

Then, all the components were mixed in a mixer for 1.5 min with rotation speed set at 140 m/min. Followed by a break for 30 s. Then the mixture was mixed at a speed of 285 m/min (a higher speed) for another 2.5 min. After mixing, the mixture was poured into 160 mm × 40 mm × 40 mm (for tensile strength at bending) and 40 mm × 40 mm × 40 mm (for compressive strength) prismatic molds and placed on a jolting machine for 1 min for vibrating compaction. The molds with samples were kept under standard conditions (a humid atmosphere—95% for 24 h (samples inside form), and (de-molded samples) kept underwater for 7, 14 and 28 days for the hydration process. The proportions of the studied cementitious composites are presented in Table 3.

### 2.4. Characterization of Surfactants Aqueous Suspensions

Surface tension in the researched solutions was determined at the processor Tensiometer produced by the KRUSS company with application of the Wilhelmy plate method, the testing temperature 25 ± 0.03 °C.

### 2.5. Characterization of CNT Aqueous Suspensions

**Figure 2:**
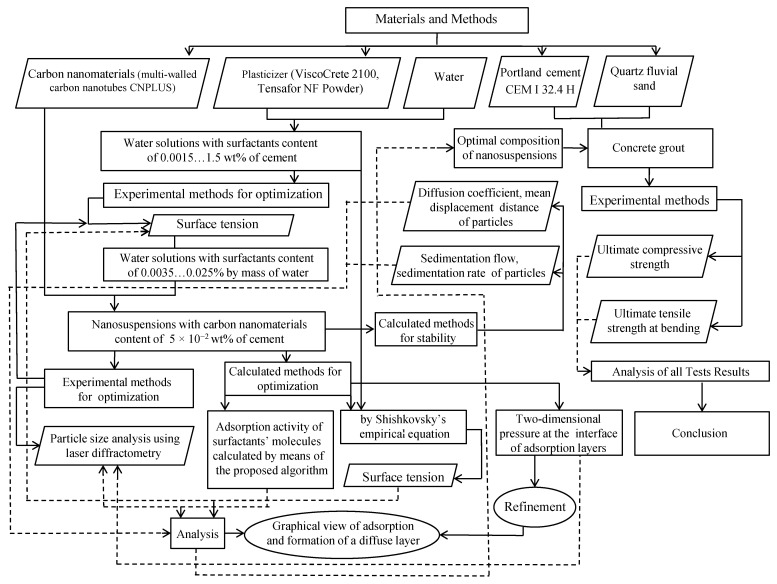
The flow chart of the research approach.

Different experimental and calculation (Figure 2) techniques were used to characterize the various parameters of the prepared CNT suspensions. Dispersive composition of carbon nanomaterials was determined by diffraction method based on the dynamic scattering of optical emission in the researched range of particles size from 0.0008 to 6.5 µm at the apparatus “Microtrac Inc.”, where a laser with a wavelength of 780 nm was used as a source of coherent monochromatic emission. Surface tension in the researched solutions was determined at the processor Tensiometer produced by the KRUSS company with application of the Wilhelmy plate method, the testing temperature being 25 ± 0.03 °C.

### 2.6. Characterization of Cementitious Composite Based on CNT Aqueous Suspensions

Flexural and compressive strength tests were carried out according to the BS EN 196-1:1995 standard. Flow values of cementitious composites were measured according to the EN 1015-3 standard. The results were obtained from the mean of 3 specimens for each test to decrease the possible errors.

The flow chart of the research approach of this study is shown in Figure 2.

## 3. Results

### 3.1. Characterization of Surfactants Aqueous Suspensions

**Figure 3:**
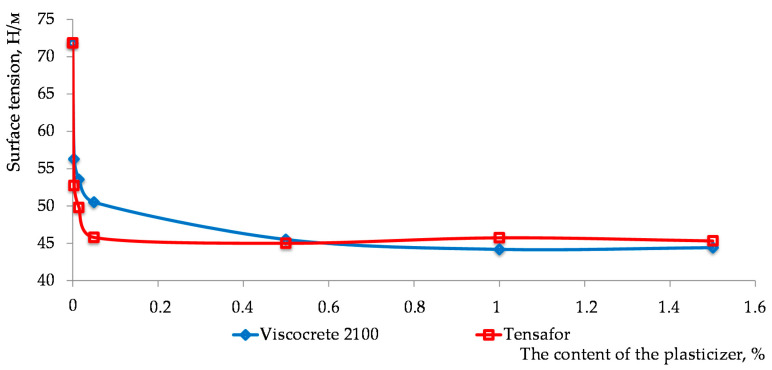
The dependency of surface tension change on the plasticizer’s concentration.

At the first stage, the percentage content of a surfactant was selected, at which effective intermolecular interaction occurs in the solution (high activity of a surfactant) resulting in the lowering of surface tension at the interface “surfactant solution–air” at a temperature of 25 °C (Figure 3).

As seen from the presented data (Figure 3), the effective content of a plasticizer does not exceed 0.05%.

### 3.2. Characterization of CNT Aqueous Suspensions

**Figure 4:**
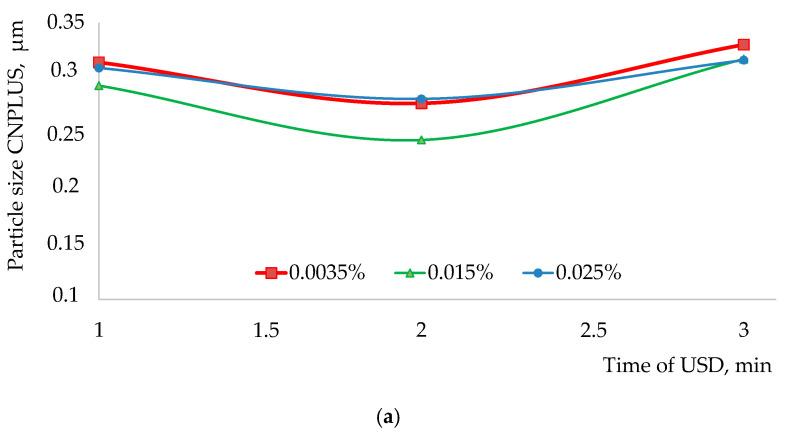

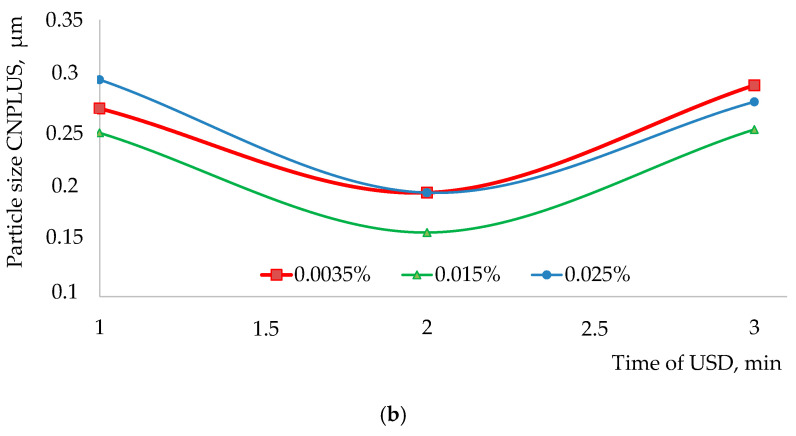
The dependency of the influence of the plasticizer’s content on the duration of ultrasonic dispersion of CNPLUS: (**a**) ViscoCrete 2100; (**b**) Tensafor.

The duration of dispersion of multi-walled carbon nanotubes (0.05%) was determined basing on the condition of minimum duration of ultrasonic treatment providing the achievement of minimum diameter of nanoparticles dispersion. The extreme points of the given effective range of plasticizers (0.0035 and 0.025%) were considered, and the results are presented in Figure 4.

From the presented information it follows that within the whole researched range (from 0.0035% to 0.025%) identical dynamic of the considered plasticizers’ impact is observed. Achievement of minimum diameter of multi-walled carbon nanotubes CNPLUS is observed after three minutes of ultrasonic dispersion that was accounted for during further experimental studies.

In Langmuir’s theory, it is assumed that the forces causing adsorption have their radius of action comparable to the molecules size; thus, the thickness of the adsorption layer does not exceed the molecules size—referring to monomolecular adsorption [47,48]. The applicability of Shishkovsky’s empirical equation (Formula (2)) to the experimental data is seen as a sign of monomolecular adsorption. For this purpose, according to the experimental dependences describing the impact of the concentration of effective surfactants’ range on surface tension of a solution, the empirical constants *A, B* and the constant related to surface tension of water $\sigma_{0}$ were calculated. The results are presented in Table 4 and Table 5.

The analysis of the presented data (Table 4 and Table 5) gives the evidence that the variation coefficient lies within 2% that demonstrates insignificant deviation of the estimated values of surface tension and applicability of Shishkovsky’s equation for describing the dependency of surface tension on the surfactant’s concentration.

The values of Γ_0_, Γ*_x_* and Γ*_f_*, calculated by means of the proposed algorithm for surfactant’s concentration equal to 0.015%, are presented in Table 6.

**Figure 5:**
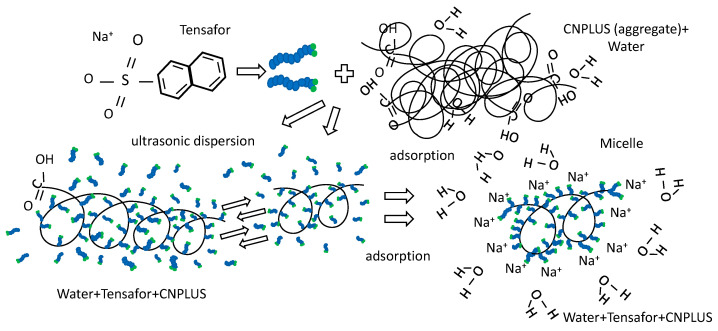
The process of adsorption and formation of a diffuse layer between the Tensafor molecules on the surface of CNPLUS.

Comparing the results of surfactants’ molecules impact on the average reduced diameter of CNPLUS (Figure 4) with the data on specific adsorption activity (Table 6), the conclusion can be drawn that, in water, the researched multi-walled nanotubes doped with carboxyl group comprising two functional groups: carbonyl (C=O) and hydroxyl (OH), are able to form hydrogen bonds. Due to this, their surfaces can interact with water, forming hydrated layers. A synergetic effect is observed when cation-active surfactant Tensafor, representing the compound of potassium naphthalene-sulfonate, is used; under the cavitation, its molecules are adsorbed on the hydrated particles CNPLUS blocking their double electric layers and simultaneously forming its own diffuse layer, less active as opposed to the initial one (Figure 5). In this case, bringing specific surface energy at the interface “surfactant’s solution–solid surface” down to a rather small value and the presence of the adsorption layer could also cause spontaneous dispersion without any external mechanical impacts.

This almost spontaneous dispersion, also known as peptization, leads to significant augmentation of the number of free particles in the volume unit that can aggregate into a spatial structure, provided that their total area is not fully stabilized, i.e., protected from coagulation. In the considered test, the peptization process is initiated after ultrasonic dispersion due to the fact that adsorption layers of water, a strong surfactant for hydrophilic surfaces, migrate along the inner surface towards the spot contacts of multi-walled CNT particles (CNPLUS), overcoming adhesion at the point of contact.

Two-dimensional pressure *Ps* is estimated under the following formula:

(9) $$P_{s}=\sigma_{0}-\sigma \left(\Gamma \right)=bx=RT\Gamma_{m}\cdot \frac{\Gamma_{f}}{\Gamma_{m}}=RT\Gamma_{f},\;$$

where Γ*_f_*—specific adsorption activity at the interface “multi-walled CNT–surfactant (0.015%)”, mol/m^2^. The results of calculations are presented in Table 7.

**Figure 6:**
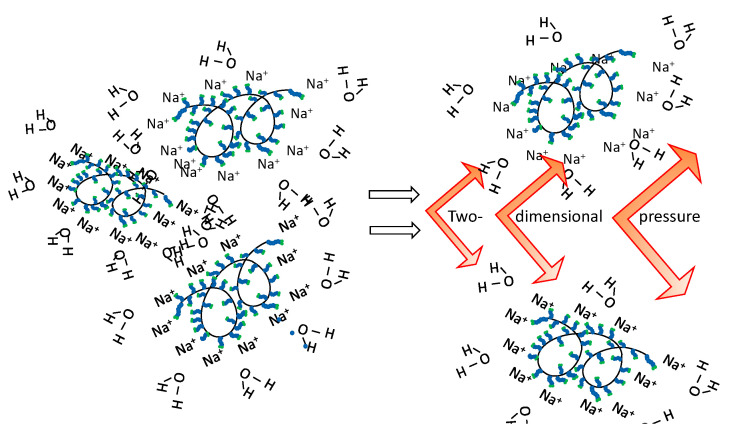
Two-dimensional pressure at the boundary of adsorption layers in dispersive system “CNPLUS–surfactant Tensafor”.

The two-dimensional pressure arising at the interface of adsorption layers in the dispersive medium with the surfactant Tensafor, being the heat load per the area unit, is sufficient to overcome adhesion on the small area of localized particles contact that leads to peptization, as presented in Figure 6.

**Figure 7:**
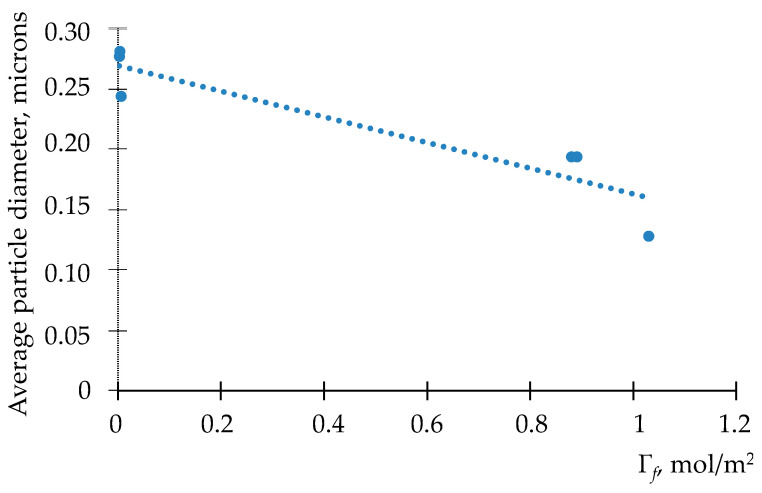
Dependency of the CNPLUS size on the value of specific adsorption on the surface in the system “CNPLUS–surfactant”.

In the dispersive system with the surfactant ViscoCrete-2100, insignificant pressure 15.6 J/m^2^ (Table 5) is fixed that preconditions low aggregate stability of such colloids. This is confirmed by the experimental data showing the dependency of mean diameter of particles of carbon nanotubes CNPLUS on the adsorption value at the interface in the system “CNPLUS–surfactant” (Figure 7).

The value of the coefficient of linear correlation is equal to *r* =−0.92, which exhibits applicability of the value Г*_f_* for predicting both effectiveness of dispersion and aggregate stability of the obtained dispersive systems.

Stability of the obtained suspensions was evaluated by sedimentation stability, i.e., the ability of the system to retain permanent volumetric distribution of particles. Sedimentation stability could be achieved when diffusion (Brownian agitation) dominates over the sedimentation process.

The amount of substance diffusing through the area unit is defined by the diffusion coefficient, under Einstein’s equation:

(10) $$D=\frac{kT}{3\pi \eta d},$$

where *d*—particle’s diameter, m; *T*—temperature, K; k—Boltzmann’s constant (1.386 × 10^−23^ J·K^−1^); *η*—viscosity of the medium, Pa·sec.

The average distance Δ*x* passed by a particle over the time t is equal to:

(11) $$\Delta x^{2}=2Dt,$$

The results of the calculation of diffusion coefficient and mean particles displacement in the aqueous solution of the surfactant at various time points of ultrasonic dispersion are presented in Table 8.

The obtained data (Table 8) prove high mobility of particles of the dispersive phase CNPLUS, their displacement on the distances far exceeding their own sizes that should have a positive impact on sedimentation stability (provided not frequent particles collision).

In order to confirm the obtained results, the stability was evaluated according to the dynamics of change of sedimentation rate *U*_ceд_:

(12) $$U_{\mathrm{ce}д}=\frac{2gr^{2}{(\rho -\rho }_{0})}{9\eta },$$

where *r*—the radius of spheric particle CNPLUS, m; *ρ*—the density of the particle CNPLUS material, kg/m^3^; *ρ*_0_—the density of aqueous solution of the surfactant, kg/m^3^; *g*—free-fall acceleration, m/s^2^, *η*—viscosity of the aqueous solution of the surfactant, Pa·sec, and specific sedimentation flow *i*_ceд_:

(13) $$i_{\mathrm{ce}д}=U_{\mathrm{ce}д}\cdot \nu \;$$

where ν—concentration of particles CNPLUS in dispersive medium.

The results of the calculations of specific sedimentation flow and sedimentation rate are outlined in Table 9.

The presented data (Table 8 and Table 9) give the evidence that, at the duration of cavitation treatment of suspensions CNPLUS in the aqueous solution of the Tensafor surfactant equal 3 min, diffusion flow (3.17 × 10^−13^) exceeds sedimentation rate (3.10 × 10^−13^). This demonstrates the sedimentation stability of such suspensions. The dispersive system “CNPLUS*–*ViscoCrete 2100*–*water” does not possess sedimentation stability.

### 3.3. Characterization of Cementitious Composite Based on CNT Aqueous Suspensions

#### 3.3.1. Mortar Past Consistory

**Figure 8:**
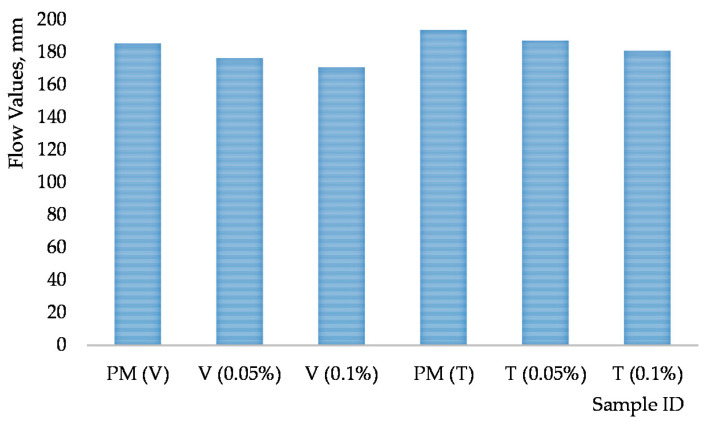
Flow values of cementitious composites.

Workability is an important parameter that evaluates the transportability of cementitious composites. The flow values for the studied cementitious composites are shown in Figure 8. According to Figure 8, CNT reduced flowability by 5%, 8%, (ViscoCrete 210) 4% and 7% (Tensafor), respectively, compared to the plain mortar. It should be noted that the content of carbon nanotubes always reduces the flowability of cementitious composites [49,50]. The significant surface areas of the carbon nanotubes are conducive to the adsorption of water molecules, which leads to a significant reduction in the content of free water and thus to a reduction in fluidity [49,50].

Previously, Parveen S et al. [50] in her study found that content of multi-walled carbon nanotubes leads to the flowability reduction around 3% for a cementitious composite with a water to cement ratio of 0.5. MWCNT were dispersed by Pluronic F-127. Du et al. [51] also indicated a 17% flowability reduction for cementitious composites containing 1% *w/c* GNP and 7.5% polycarboxylate superplasticizer with a 0.5 *w*/*c* ratio.

#### 3.3.2. Mechanical Properties

**Figure 9:**
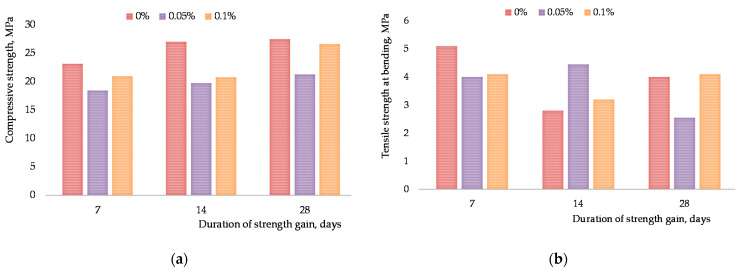
Influence of the CNPLUS and ViscoCrete 2100 content on the strength properties of concrete grout: (**a**) ultimate compressive strength; (**b**) ultimate tensile strength at bending.

**Figure 10:**
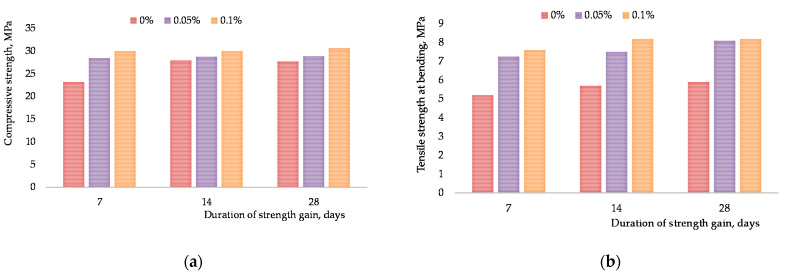
Influence of the CNPLUS and Tensafor content on the strength characteristics of concrete grout: (**a**) ultimate compressive strength; (**b**) ultimate tensile strength at bending.

After mixed design optimization for the CNTs aqueous suspension, the impact was established of the revealed superficial phenomena occurring at the interface in the system “primary carbon materials aqueous solutions of surfactants” on finite properties of composite (ultimate compressive strength and ultimate tensile strength at bending). The results for the cementitious composites reinforced by different CNTs concentrations are shown in Figure 9 (0.5 *w*/*c* ratio) and Figure 10 (0.45 *w*/*c* ratio) (after 7, 14 and 28 days of the hydration period), including their corresponding standard deviations.

The analysis of Figure 9a,b shows that insertion of CNPLUS does not increase ultimately compressive strength. Irregular distribution in aqueous medium with ViscoCrete 2100 (visible particles setting) is reflected on statistical variability of the data on ultimate compressive and bending strength. Agglomeration CNT reduced flexural and compressive strength, which is consistent with previous studies [52]. Standard deviations are bigger than 15% for all samples.

The analysis of Figure 10a demonstrates the increase in compressive strength only at the early stage of hardening. At the age of 7 days, the increment of strength reaches 30%, which is later leveled off when the design strength is achieved, i.e., the difference between the design strength of a concrete grout specimen with nano-modifier and the non-modified one does not exceed 5%, which is within measurement accuracy. When investigating the results of measuring tensile strength at bending (Figure 10b), significant influence of evenly distributed and dispersed particles CNPLUS in the aqueous medium with Tensafor was revealed. Thus, design tensile strength at bending for the specimens of concrete grout with the nano-modifier exceeds the strength of the non-modified concrete specimens by 38%. Standard deviations are less than 1.6% for all samples. Tensafor provides the formation of a micellar structure in coagulation layers [53], which is a structural–mechanical barrier with an external hydrophilic surface. It happened due to the bonding the was established between the CNTs and cement matrix. The CNT-modified cement can mitigate the propagation of matrix micro-cracks by forming CNT bridges over the incipient micro-crack gaps [20,52,53,54,55]. The load borne by the cement matrix is effectively shifted by strong bonds to the CNTs.

## 4. Conclusions

In the present study, the interfacial phenomena at the interface in the system “Carbon primary materials–water solutions of surfactants” for cement materials were investigated. Moreover, Tensafor was successfully used to obtain a high-quality dispersion of a high concentration CNTs aqueous suspension. Using this route, we studied the influence of the surfactant with added CNTs concentration, sonication time and surface adsorption activity.

The results of laser spectroscopy, CNTs bundle size measurement and specification adsorption activity of surfactants’ molecules, diffusion coefficient, sedimentation flow and sedimentation rate showed that using the soap-like surfactants, with the compound potassium naphthalene sulfonate (Tensafor), resulted in a highly homogeneous aqueous dispersive medium with full stabilization of CNTs. The micelle-like structure in the coagulation layers forms a structural and mechanical barrier with the external hydrophilic surface.

Applicability of Shishkovsky’s empirical equation for describing surface activity of the primary carbon material CNPLUS in the plasticizer’s aqueous solution was confirmed. The variation coefficient does not exceed 2%, which proves the insignificant deviation of the estimated values of surface tension from the data obtained experimentally. However, it is worth mentioning that its applicability should be double-checked for other possible scenarios.

Adsorption and structural constituent parts play crucial role in stabilization of dispersive media. It was stated that with the growth of adsorption of the surfactant Tensafor on the solid surface of CNPLUS arising due to auto-adhesive strength, the intensity of particles’ dispersion also increases. Thus, in the system “CNPLUS–aqueous solution of Tensafor”, the minimum particles diameter of 128 nm for the nano dispersive phase is observed at the maximum value of specific adsorption is 1030 mmol/m^2^. In the system “CNPLUS–aqueous solution of ViscoCrete 2100”, the minimum size of the particles for nano-dispersive phase is also fixed at 244 nm, when the maximum value of specific adsorption accounts for 6.3 mmol/m^2^. For the considered dispersive systems, we obtained the correlation dependency of the mean diameter of particles for carbon nanotubes CNPLUS on the value of adsorption at the interface in the system “CNPLUS–surfactant”. The value of the coefficient of linear correlation for the obtained dependency is equal ***r*** = −0.92, which shows the applicability of the value Γ*_f_* for predicting both effectiveness of dispersion and aggregate and sedimentative stability of the obtained dispersive systems.

It was stated that two-dimensional pressure arising at the interface of adsorption layers in the dispersive medium with the surfactant Tensafor 2553.2 J/m^2^ is sufficient to overcome adhesive strength at a small area of the localized contact of carbon nanoparticles CNPLUS, which explains the peptization and stabilization of particles’ surface. In the dispersive system with the surfactant ViscoCrete-2100, an insignificant pressure demonstrating low aggregate stability of such colloids, was observed. Despite these findings, the exact causal mechanism behind these trends is not well understood.

It was proven that insertion of CNPLUS in the aqueous solution with ViscoCrete 2100 into the concrete grout specimens does not affect ultimate compressive strength, whereas irregular distribution of CNPLUS (visible particles setting) led to statistical variability of the data on ultimate compressive and bending strength. Significant influence of evenly distributed and dispersed particles CNPLUS in the aqueous medium with Tensafor was revealed. This compound provides the formation of a micellar structure in coagulation layers, which is a structural–mechanical barrier with an external hydrophilic surface. Design tensile strength at bending for the concrete grout made with the use of the stable colloid system “CNPLUS–aqueous solution of Tensafor” exceeds the strength of concrete grout specimens without nano-modifiers by 38%. The results obtained also showed 0.05% as an optimum concentration of CNT. The outcomes of the present study illustrate the practical application potential for cement-based materials.

## Figures and Tables

**Table 1:** Plasticizers’ characteristics.

Property	ViscoCrete 2100	Tensafor
pH (Sol. 10%)	4.5	7.0
Solubility (in water), %	-	total hasta 40%
Humidity, % Molecular weight, g/mol	– –	10 max 345.5
Density, g/cm^3^	1.08	–

**Table 2:** CNPLUS characteristics.

Property	CNPLUS
Specific surface area, m^2^/g	>110
Density, g/cm^3^	0.14
Color	black
Outside Diameter, nm	10–20
Length, µm	10–20
Carbon content, %	98
-OH content, wt%	2.48
Ash, wt%	<5
Part number	GMC355

**Table 3:** Mix proportions of the CNTs-cementitious composites.

Sample ID	CNT, wt% of Cement	Cement, g	W/C Ratio	Sand/Cement Ratio
Plain Mortar (V)	0	450	0.5	3
V (0.05%)	0.05	450	0.5	3
V (0.1%)	0.1	450	0.5	3
Plain Mortar (T)	0	450	0.45	3
T (0.05%)	0.05	450	0.45	3
T (0.1%)	0.1	450	0.45	3

**Table 4:** Evaluation of applicability of Shishkovsky’s empirical equation for description of the impact of the ViscoCrete-2100 concentration on the surface tension of aqueous solutions.

Surfactant’s Concentration, %	Experimental Value	Calculated Value			
	Surface Tension, σ, H/м	Constant	Surface Tension, σ, H/м	Mean-Square Deviation	Variation Coefficient, %
0	71.80	$\sigma_{0}$ = 71.8	71.80	0.000	0.00
0.0035	56.28	*B* = 2.04	56.11	0.014	0.03
0.0075	53.95		54.56	0.184	0.34
0.0150	53.57	*A* = 1.6 × 10^−6^	53.14	0.091	0.17
0.0250	51.90		52.10	0.020	0.04

**Table 5:** Evaluation of applicability of Shishkovsky’s empirical equation for description of the impact of the Tensafor concentration on the surface tension of aqueous solutions.

Surfactant’s Concentration, %	Experimental Value	Calculated Value			
	Surface Tension, σ, H/м	Constant	Surface Tension, σ, H/м	Mean-Square Deviation	Variation Coefficient, %
0	71.84	$\sigma_{0}$ = 71.8	71.83	0.000	0.00
0.0035	52.73	*B* =3. 43	53.83	0.606	1.14
0.0075	52.51		51.23	0.824	1.59
0.0150	49.79	*A* = 1.8 × 10^−5^	48.85	0.439	0.89
0.0250	45.83		47.10	0.810	1.74

**Table 6:** Specific adsorption activity of surfactants’ molecules.

Value of Adsorption at Different Phase Interfaces, mol/m^2^	ViscoCrete 2100	Tensafor
“aqueous surfactant solution–air”, Γ_0_, mol/m^2^	73.1470	123.0586
“aqueous surfactant solution–air” after the insertion of CNPLUS, Γ*_x_*, mol/m^2^	73.1409	122.0317
“CNPLUS–surfactant” Г*_f_*, mol/m^2^	6.3 × 10^−3^	1.026

**Table 7:** Two-dimensional pression at the interface “CNPLUS–surfactant (0.015%)”.

Parameter	ViscoCrete-2100	Tensafor
Two-dimensional pressure at the interface of adsorption layers, J/m^2^	15.6	2553.2

**Table 8:** Diffusion coefficient and mean displacement distance for the CNPLUS particles in the aqueous solution of the surfactant.

Surfactant Type	Duration of Ultrasonic Dispersion, Min				
	1	2	3	4	5
ViscoCrete 2100	1.75 × 10^−14^ 1.12 × 10^−5^	3.60 × 10^−14^ 1.61 × 10^−5^	6.97 × 10^−14^ 2.24 × 10^−5^	8.17 × 10^−14^ 2.42 × 10^−5^	8.50 × 10^−14^ 2.47 × 10^−5^
Tensafor	2.27 × 10^−14^ 1.28 × 10^−5^	4.51 × 10^−14^ 1.80 × 10^−5^	3.17 × 10^−13^ 4.78 × 10^−5^	1.85 × 10^−13^ 3.65 × 10^−5^	2.00 × 10^−13^ 3.80 × 10^−5^

Note. Numerator—the values of diffusion coefficient, m^2^/s; denominator—mean particles displacement, m; temperature dependency of medium’s viscosity η = 0.374·e^−0.043T^.

**Table 9:** Specific sedimentation flow and sedimentation rate in the system “CNPLUS–surfactant–water”.

Surfactant Type	Duration of the Ultrasonic Dispersion, min				
	1	2	3	4	5
ViscoCrete 2100	4.26 × 10^−12^ 2.7 × 10^−2^	4.72 × 10^−12^ 3.00 × 10^−2^	5.11 × 10^−12^ 3.20 × 10^−2^	1.28 × 10^−11^ 8.10 × 10^−2^	3.08 × 10^−11^ 1.94 × 10^-1^
Tensafor	2.63 × 10^−12^ 1.7 × 10^−2^	3.57 × 10^−12^ 2.3 × 10^−2^	3.10 × 10^−13^ 2.00 × 10^−3^	3.11 × 10^−12^ 2.0 × 10^−2^	7.14 × 10^−12^ 4.50 × 10^−2^

Note. Numerator—the values of specific sedimentation flow, particles/cm; denominator—sedimentation rate, cm/year.

## Data Availability

Data are contained within the article.
